# Old tale new admirers, cetuximab maintenance in metastatic colorectal cancer: a systematic review and meta-analysis

**DOI:** 10.3389/fphar.2026.1845800

**Published:** 2026-06-03

**Authors:** Yici Yan, Zhenyi Lin, Jing Yuan, Weidong Gao, Chenxu Jiao, Leitao Sun, Xun Sun

**Affiliations:** 1 The First Affiliated Hospital of Zhejiang Chinese Medical University (Zhejiang Provincial Hospital of Chinese Medicine), Hangzhou, China; 2 Academy of Chinese Medical Science, Zhejiang Chinese Medical University, Hangzhou, China; 3 Key Laboratory of Neuropharmacology and Translational Medicine of Zhejiang Province, School of Pharmaceutical Sciences, Zhejiang Chinese Medical University, Hangzhou, China; 4 Longyou Hospital of Chinese Medicine, Quzhou, Zhejiang, China

**Keywords:** cetuximab, maintenance, meta-analysis, metastatic colorectal cancer, overall survival, progression-free survival

## Abstract

**Background:**

metastatic colorectal cancer (mCRC) represents a major global burden and the decision on whether to pursue maintenance with cetuximab (CET) remains controversial. Therefore, this meta-analysis was performed to assess the efficacy and safety of CET maintenance therapy.

**Methods:**

After literature search of PubMed, Embase, Cochrane Library and Scopus from inception to March 2026, two investigators selected eligible studies and extracted relevant data. The key outcomes were progression-free survival (PFS) and overall survival (OS). A random-effects model was used for pooling. The relative risks (RRs) with 95% confidence interval (CI) of all-grade and high-grade (≥3) adverse effects (AEs) were used to study its safety. Heterogeneity was also investigated through subgroup and sensitivity analysis.

**Results:**

A total of 6 studies with 727 patients were included. CET maintenance therapy prolonged the PFS (HR: 0.42, 95% CI: 0.27 to 0.57, P < 0.01) and OS (HR: 0.47, 95% CI: 0.19 to 0.74, P < 0.01) of mCRC patients. Subgroup analysis showed that Asian patients (HR: 0.53, 95% CI: 0.15 to 0.60, P = 0.010) received more survival benefit from CET maintenance therapy than non-CET therapy or observation. Although diarrhea and rash were more common, the RR of all-grade AEs revealed no statistical significance.

**Conclusion:**

CET maintenance therapy may be associated with improved outcomes compared with observation, especially in Asian patients. But it may slightly increase treatment-related toxicities. Therefore, subsequent large sample, multi-center randomized controlled trials (RCTs) are needed to further verify our conclusions.

**Systematic Review Registration:**

https://www.crd.york.ac.uk/prospero/display_record.php?ID=CRD42024513879, identifier CRD42024513879.

## Introduction

Colorectal cancer constitutes a significant global health concern since it is the third most common malignancy and the second leading cause of cancer death worldwide, responsible for almost 920,000 deaths every year (Sung et al., 2021). Approximately 25% of patients manifest metastatic disease at the time of diagnosis, and nearly half will subsequently develop metastases during the course of their illness (Biller and Schrag, 2021). The mainstay of treatment for metastatic colorectal cancer (mCRC) involves a combination of oxaliplatin or irinotecan with a fluoropyrimidine (fluorouracil or capecitabine), along with leucovorin (LV) (Morris et al., 2023). Notably, the efficacy of chemotherapy is further enhanced when combined with cetuximab (CET) (Van Cutsem et al., 2009).

CET is an epidermal growth factor receptor (EGFR) inhibitor primarily employed in the management of mCRC and head and neck cancer (Goldberg and Kirkpatrick, 2005). The combination of FOLFOX (fluorouracil, oxaliplatin, and LV) or FOLFIRI (fluorouracil, irinotecan, and LV) with CET is regarded by major guidelines as a safe and effective first line therapeutic option for RAS wild-type left-sided mCRC (Morris et al., 2023; CervantesA et al., 2023). For the 50% of patients diagnosed with Kirsten rat sarcoma viral oncogene homolog (KRAS)/Neuroblastoma RAS viral oncogene homolog (NRAS)/v- -Raf murine sarcoma viral oncogene homolog B (BRAF) wild-type mCRC, these combinations can prolong median survival by 2–4 months when compared with chemotherapy alone (Biller and Schrag, 2021). However, the optimal duration of first-line induction chemotherapy following the attainment of maximum response remains controversial due to potential toxic effects, such as oxaliplatin-induced neuropathy and irinotecan-associated chronic fatigue (Cunningham et al., 2010). This has prompted consideration of maintenance treatment with “lighter therapy” as an alternative to the conventional approach of continuing induction treatment until progression. Since CET is active as a single-agent therapy in RAS wild-type mCRC, it may allow chemotherapy-free intervals with lower toxicity.

To date, 2 randomized controlled trials (RCTs) have demonstrated the feasibility of maintenance with CET maintenance and its association with prolonged progression-free survival (PFS), overall survival (OS), and chemotherapy-free intervals (Boige et al., 2023; Cremolini et al., 2018a). However, the level of evidence in favor of maintenance therapy with CET is not robust due to the limited number of the existing RCTs and the variability in regimens employed. Therefore, a meta-analysis was carried out to evaluate the efficacy and safety of CET as maintenance therapy after induction chemotherapy for mCRC patients.

## Methods

### Search strategy

This analysis was performed according to the Preferred Reporting Items for Systematic Reviews and Meta-Analyses (PRISMA 2020) statement (Page et al., 2021). To evaluate the efficacy of maintenance treatment with CET for patients with mCRC after the initial induction chemotherapy, PubMed, Embase, Cochrane Database and Scopus were searched from inception to March 2026 by two independent investigators. The following search term ‘Colorectal Neoplasms’ AND ‘Cetuximab’ was employed without restrictions on country or language and both MeSH terms and free-text terms were used. The complete search string was introduced in Supplementary Table S1. Additional records identified through other sources, including references from the reviews, were also checked to ensure that all eligible articles were collected. The protocol of our study has been registered in PROSPERO https://www.crd.york.ac.uk/prospero/display_record.php?ID=CRD42024513879 (CRD42024513879).

### Study selection

The inclusion criteria were as follows: (1) patients with mCRC who received CET-containing induction therapy; (2) the study compared maintenance therapy between a CET arm and a non-CET arm or observation arm; (3) the control group underwent the same initial induction chemotherapy as the interventional group; (4) outcome included PFS or OS. (5) eligible studies comprised clinical trials or observational studies.

Simultaneously, articles were excluded if they met any of the following conditions: (1) studies not published as full-text articles; (2) data in the article were unavailable, such as cases where the article presented median PFS and OS without 95% confidence interval (CI) or Kaplan-Meier analysis; (3) studies involved the same group of participants; in cases of multiple publications related to the same study, the most updated version was chosen; (4) excluded article types were reviews, meta-analyses, letters, editorials, protocols, and irrelevant studies, such as laboratory articles.

### Data extraction

Using a standardized predesigned data extraction form, two investigators independently extracted data from the included articles. Any disparities among investigators were resolved through discussion. The collected data included: (1) study characteristics: authors, publication year, countries, study types, regimens, number of patients, median follow-up durations, median age, etc. (2) outcomes: hazard ratio (HR) with 95% CI for PFS and OS, adverse events (AEs) of all grade and ≥ grade 3.

### Quality assessment

According to the study type, the included articles were assessed in two parts.

The quality of the clinical trial was assessed according to the Cochrane evaluation handbook of RCTs, including selection bias (randomization sequence generation), selection bias (allocation concealment), performance bias (blinding of participants and personnel), detection bias (blinding of outcome assessment), attrition bias (incomplete outcome data), reporting bias (selective reporting), and other biases. Each bias item was defined as ‘Low Risk’, ‘High Risk’ or ‘Unclear Risk’ by two independent investigators. RevMan 5.4 was used for this section, and consensus was reached by a third author.

Meanwhile, cohort studies underwent assessment utilizing the Newcastle-Ottawa Scale (NOS), which employed a star rating system to assess research across three domains: participant selection, comparability of study groups, and adequacy of results and follow-up. Each of these criteria is assigned a specific number of stars, and the total number of stars indicates the overall quality of the study. A higher star rating suggests a lower risk of bias and better study quality. Studies were categorized with a score of 7–9 stars as low risk of bias, those with a score of 4–6 stars as moderate risk, and those with a score of 0–3 stars as high risk of bias. The overall quality of evidence was assessed using the GRADE methodology.

### Statistical analysis

Statistical analyses of study outcomes were performed and pooled as forest plots by Stata 18.0. The HR with 95% CI was used to assess PFS and OS. HR < 1 favored the intervention group, while HR > 1 favored the control group. RR with 95% CI was used to analyze AEs. RR < 1 suggested higher toxicity in the control group, whereas RR > 1 indicated the opposite. Chi-square Q test and I^2^ statistic were used to assess statistical heterogeneity. I^2^<30% represented low heterogeneity, 30% ≤ I^2^ ≤ 60% indicated moderate heterogeneity, and I^2^>60% revealed high heterogeneity. Due to the diversity of study designs and differences in interventions, the random‐effects model was used to improve statistical reliability. Funnel plots were used to measure potential publication bias. Sensitivity analysis was performed to estimate the robustness of the findings. All reported P-values were two-sided and statistically significant when P < 0.05.

## Results

### Study selection

**FIGURE 1:**
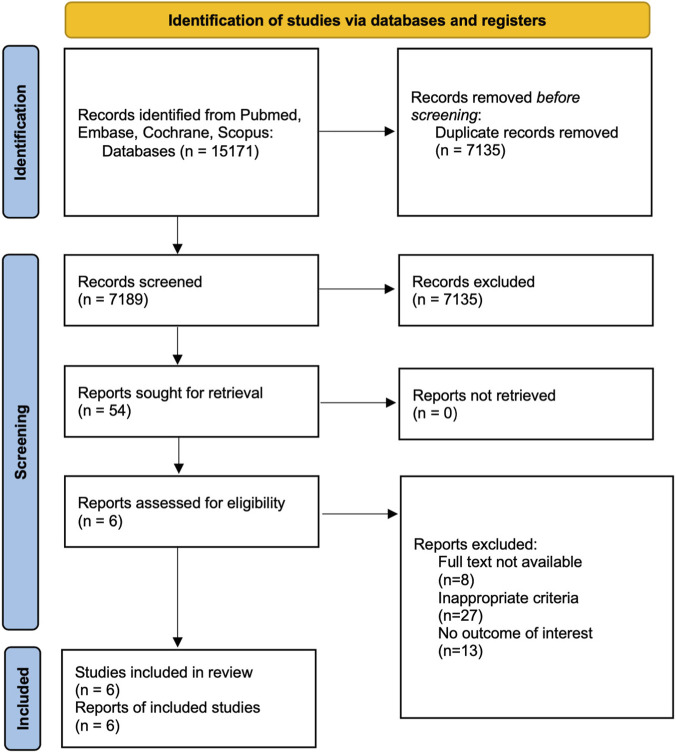
Flow chart of study selection.

In this study, 15,171 relevant articles were initially retrieved according to the search strategy. After removing duplications, 7,189 articles were included. By browsing titles and abstracts, 7,135 articles were excluded while 54 were obtained. After a detailed examination of the full text, 48 articles were eliminated. Among the excluded studies, 8 studies were unavailable in full text, 27 did not meet appropriate criteria, and 13 lacked the outcome of interest. Ultimately, 6 studies were included for the final analysis (Boige et al., 2023; Cremolini et al., 2018a; Chen B. et al., 2020; Jiang et al., 2020; Li et al., 2024; Yuan et al., 2021). The specific screening steps are summarized in Figure 1.

### Study characteristics

In total, 727 patients were enrolled in this study. Three studies used FOLFIRI + CET for first-line induction chemotherapy, 2 used FOLFOXIRI + CET, one used FOLFIRI + CET/mFOLFOX6+CET. In maintenance treatment, three studies used CET monotherapy in the intervention group, whereas the remaining 3 used the combination therapy. Among these, one 1 used CET + capecitabine, 1 employed CET + irinotecan and 1 utilized CET + chemotherapy. In 3 studies, patients were assigned to the observation group as the control. In the remaining 3 studies, 2 used bevacizumab as control group and 1 used capecitabine. Four studies were from China, 1 was from France and 1 was from Italy. All 6 studies reported the PFS, and 5 studies reported the OS. The detailed characteristics of the included studies were summarized in Table 1 and Supplementary Table S2.

**TABLE 1:** Characteristics of included studies.

Author, year	Country	Study type	Median follow-up	No. of patients	First-line induction chemotherapy	Intervene	Control	HR (95% CI) for PFS	HR (95% CI) for OS
Boige V, 2023	France	RCT	40.5mo	139	FOLFIRI + CET	CET (500 mg/m^2^, q2w)	Observation	0.46 (0.32–0.67)	0.84 (0.50–1.44)
Chen B, 2020	China	Cohort	27.0mo	222	FOLFOXIRI + CET	CET (500 mg/m^2^, q2w)	BEV (5 mg/kg, q2w)	0.31 (0.15–0.46)	0.22 (0.11–0.37)
Cremolini C, 2018	Italy	RCT	44.0mo	78	FOLFOXIRI + CET	CET (500 mg/m^2^, q2w)	BEV (5 mg/kg, q2w)	0.73 (0.46–1.17)	0.98 (0.52–1.87)
Jiang T, 2020	China	Non-RCT	NA	72	FOLFIRI + CET	CET + CPT-11	Observation	0.20 (0.11–0.37)	0.21 (0.12–0.38)
Li J,2023	China	Cohort	NA	39	FOLFIRI + CET	CET (500 mg/m^2^ on d1, q2w) +CAP (1 g/m^2^ bid on days 1–14, q3w	CAP (1 g/m^2^ bid on days 1–14, q3w)	0.40 (0.20–1.00)	NA
JY M, 2021	China	Cohort	NA	177	FOLFIRI + CET/mFOLFOX6+CET	CET/CET + Chemo	Observation	0.58 (0.41–0.82)	0.57 (0.36–0.91)

NA, not available; No, number; FOLFIRI, Fluorouracil + Irinotecan + Leucovorin; FOLFOXIRI, Fluorouracil + Oxaliplatin + Irinotecan + Leucovorin; CET, cetuximab; q2w, every 2 weeks; CPT-11, Irinotecan; q3w, every 3 weeks; CAP, capecitabine; Chemo, Chemotherapy; BEV, bevacizumab; HR, hazard ratio; CI, confidence interval; PFS, Progression-Free Survival; OS, overall survival.

### Quality assessment

According to the Cochrane Collaboration tools, all included trials (2 RCTs and 1 Non-RCT) were of low risk of bias (Supplementary Figures S1, S2). Regarding selection bias (random sequence generation), 2 study was assessed as “low risk” while 1 as “unclear risk”. In terms of allocation concealment, all studies were classified as “unclear risk”. In terms of performance bias, all studies were evaluated as “unclear risk”. In terms of detection bias, 2 studies were at “low risk” and 1 was at “unclear risk”. Regarding attrition bias, all studies were classified as “low risk”. All studies were rated as “low risk” for reporting bias. And all studies were labeled “low risk” for other biases.

According to the NOS criteria, the three cohort studies were evaluated respectively with 6, 5, and 4 stars. All studies were clarified as moderate risk, with a mean score of 5, which indicated the overall bias was not high and the detailed assessments can be found in Supplementary Table S3.

According to the GRADE assessment, the quality of evidence was moderate for both OS and PFS (Supplementary Table S4).

### Efficacy

#### PFS

**FIGURE 2:**
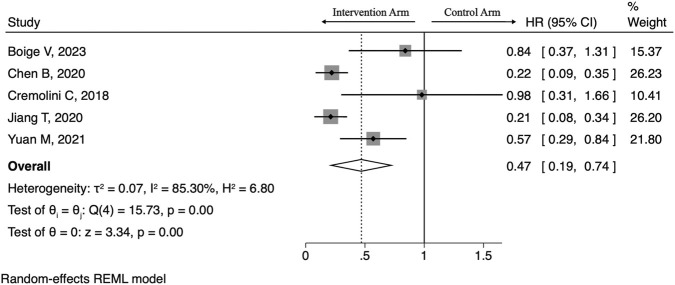
PFS analysis of cetuximab on metastatic colorectal cancer.

PFS data were obtained from a collective of 727 patients across all studies. The significant PFS improvement observed with CET maintenance was confirmed. The risk of progression or death was reduced by 58% with CET maintenance versus non-CET therapy or observation (HR: 0.42, 95% CI: 0.27 to 0.57, P < 0.01, Figure 2). At the same time, random-effects model was chosen for PFS analysis since the heterogeneity was high (I^2^ = 68.94%).

#### OS

**FIGURE 3:**
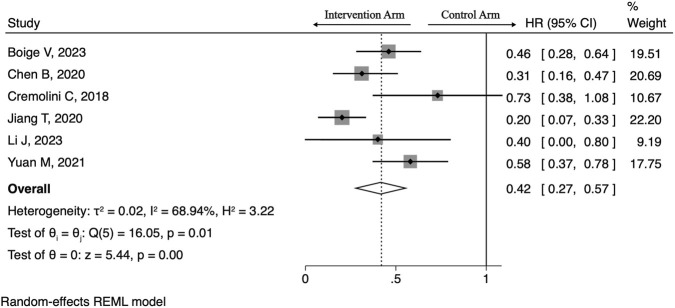
OS analysis of cetuximab on metastatic colorectal cancer.

Five studies encompassing 688 patients provided OS data. According to OS analysis, the risk of progression or death was reduced by 53% with CET maintenance versus non-CET therapy or observation (HR: 0.47, 95% CI: 0.19 to 0.74, P < 0.01, Figure 3), indicating that patients treated with CET maintenance therapy received more survival benefits in prolonging life. The heterogeneity among the studies was high (I^2^ = 85.30%). Therefore, a random effects model was used to calculate the pooled HR.

### Subgroup analysis

To further examine data heterogeneity, subgroup analyses were performed according to location, study type, regimen of induction chemotherapy, regimen of experimental group and control group (Table 2). OS benefit was observed in Asian patients (HR: 0.30, 95% CI: 0.15 to 0.60, P = 0.010), but not in European patients (P = 0.591). PFS benefit was observed in both Asian patients (HR: 0.36, 95% CI: 0.21 to 0.59, P < 0.001), and European patients (HR: 0.57, 95% CI: 0.36 to 0.89, P = 0.013). The OS benefit with CET maintenance was observed in patients from cohort (HR: 0.36, 95% CI: 0.14 to 0.92, P = 0.032), but not in RCT (P = 0.591). PFS benefit was observed in patients from both RCT (HR: 0.57, 95% CI: 0.36 to 0.89, P = 0.013) and cohort (HR: 0.44, 95% CI: 0.29 to 0.67, P < 0.001). PFS benefit with CET maintenance was seen in patients treated with FOLFIRI + CET as induction chemotherapy (HR: 0.37, 95% CI: 0.28 to 0.50, P < 0.001), and in patients treated with FOLFOXIRI + CET (HR: 0.51, 95% CI: 0.36 to 0.74, P < 0.001). OS benefit with CET maintenance was observed in patients treated with FOLFIRI + CET as induction chemotherapy (HR: 0.45, 95% CI: 0.31 to 0.67, P < 0.001), and in patients treated with FOLFOXIRI + CET (HR: 0.45, 95% CI: 0.29 to 0.69, P < 0.001). PFS benefit with CET maintenance was observed in both the CET monotherapy group (HR: 0.48, 95% CI: 0.31 to 0.74, P = 0.001) and the combination therapy group (HR: 0.27, 95% CI: 0.14 to 0.52, P < 0.001). PFS benefit was observed in patients treated with bevacizumab (HR: 0.49, 95% CI: 0.08 to 0.90, P = 0.030) and observation (HR: 0.40, 95% CI: 0.18 to 0.63, P < 0.001). CET maintenance showed a marginal OS benefit in studies with observation as control (HR: 0.47, 95% CI: 0.22 to 1.00, P = 0.050), while no significant benefit was observed in studies with bevacizumab as control (P = 0.302).

**TABLE 2:** Subgroup analysis of PFS and OS.

Endpoint	Subgroup	No. of studies	No. of patients	HR (95% CI)	P	I^2^
PFS	Location					
	Asia	4	217	0.36 (0.21.0.59)	<0.001	70.40%
	Europe	2	510	0.57 (0.36.0.89)	0.013	56.70%
	Study type					
	RCT	2	217	0.57 (0.36.0.89)	0.013	56.70%
	Cohort	3	510	0.44 (0.29.0.67)	<0.001	45.60%
	Regimen of induction chemotherapy					
	FOLFIRI + CET	3	250	0.37 (0.28.0.50)	<0.001	62.00%
	FOLFOXIRI + CET	2	300	0.51 (0.36.0.74)	<0.001	81.10%
	Regimen of experimental group					
	CET	3	439	0.48 (0.31.0.74)	0.001	63.50%
	Combination	2	111	0.27 (0,14,0.52)	<0.001	43.80%
	Regimen of control group					
	Bev	2	388	0.49 (0.08, 0.90)	0.030	77.86%
	Observation	3	300	0.40 (0.18, 0.63)	<0.001	81.19%
OS	Location					
	Asia	3	217	0.30 (0.15.0.60)	0.010	78.40%
	Europe	2	471	0.89 (0.59.1.34)	0.591	0.00%
	Study type					
	RCT	2	217	0.89 (0.59.1.34)	0.591	0.00%
	Cohort	2	399	0.36 (0.14.0.92)	0.032	83.30%
	Regimen of induction chemotherapy					
	FOLFIRI + CET	2	211	0.45 (0.31.0.67)	<0.001	91.50%
	FOLFOXIRI + CET	2	300	0.45 (0.29.0.69)	<0.001	90.90%
	Regimen of control group					
	Bev	2	388	0.46 (0.11, 2.00)	0.302	90.90%
	Observation	3	300	0.47 (0.22, 1.00)	0.050	83.70%

PFS, Progression-Free Survival; OS, overall survival; RCT, randomized controlled trial; FOLFIRI, Fluorouracil + Irinotecan + Leucovorin; FOLFOXIRI, Fluorouracil + Oxaliplatin + Irinotecan + Leucovorin; CET, cetuximab; Bev, Bevacizumab; No, number; HR, hazard ratio; CI, confidence interval.

### Safety

As shown in Table 3, the RR of hematologic adverse events revealed no statistical significance between the CET maintenance group and non-CET or observation group: anemia (P = 0.40) and neutropenia (P = 0.51). As for gastrointestinal adverse events, although CET maintenance resulted in a higher risk of AEs of diarrhea (P < 0.01), there was no significant difference in the RR of nausea or vomiting (P = 0.33). In terms of dermatologic adverse events, while CET maintenance therapy was more likely to cause all-grade rash (P < 0.01), no significant difference was observed in the RR of mucositis (P = 0.72). In addition, fatigue (P = 0.05) caused by CET maintenance therapy showed borderline significance.

**TABLE 3:** Treatment-related common adverse events in this meta-analysis.

Adverse events		RR (95% CI)			
		All Grade	*P* value	Grade ≥ 3	*P* value
Hematologic	Anemia	1.22 (0.77, 1.93)	0.40	NA	NA
	Neutropenia	1.35 (0.55, 3.30)	0.51	2.75 (1.16, 6.54)	0.02
Gastrointestinal	Diarrhea	3.33 (1.75, 6.32)	<0.01	1.47 (0.54, 4.02)	0.45
	Nausea or vomiting	1.90 (0.53, 6.86)	0.33	NA	NA
Dermatologic	Mucositis	1.32 (0.29, 6.12)	0.72	NA	NA
	Rash	10.99 (3.57, 33.82)	<0.01	NA	NA
Other	Fatigue	1.59 (1.01, 2.51)	0.05	NA	NA

RR, relative risk; NA, not available.

Subsequently, this study analyzed high-grade (grade≥3) adverse events to determine the severity of adverse events. Patients with CET maintenance tended to have a higher risk of neutropenia (P = 0.02). There was no significant difference in the RR of diarrhea (P = 0.45).

### Publication bias and sensitivity analysis

We used the funnel plot to determine publication bias (Supplementary Figures S3, S4). The funnel plots for both PFS and OS appeared asymmetric overall, suggesting that publication bias could not be excluded. Sensitivity analysis was performed to evaluate the reliability of the findings (Supplementary Figures S5, S6). No statistically significant changes in the overall results were observed after removing each included study, supporting the robustness of the pooled results.

## Discussion

As an EGFR inhibitor, the application of CET in mCRC remains mostly in the stage of combination therapy and first-line therapy (Van Cutsem et al., 2009; Boke et al., 2009; Cunningham et al., 2004). For example, the CRYSTAL study has revealed that the addition of CET to FOLFIRI as first-line treatment could improve survival benefit of patients with mCRC (Van Cutsem et al., 2009). Similarly, the OPUS demonstrated that the combination of CET and FOLFOX, as compared with FOLFOX alone, improved objective response rate (ORR) in mCRC (Boke et al., 2009). In our study, more clinical benefits from CET as maintenance therapy were revealed after statistical analysis of relevant data of 727 patients from 6 enrolled RCTs and cohorts. Concretely speaking, CET maintenance was associated with improved PFS and OS for mCRC when compared with other non-CET maintenance or observation, especially in Asian patients and patients treated with FOLFOXIRI + CET as induction chemotherapy.

Subgroup analysis has demonstrated PFS and OS benefit in mCRC patients in both induced therapy subgroups: FOLFOXIRI + CET and FOLFIRI + CET. This may suggest that CET maintenance therapy provides significant clinical benefits regardless of the induction regimen used. The Gruppo Oncologico Nord Ovest (GONO) trial highlighted the advantages of the FOLFOXIRI regimen, showing improved response rates, PFS, and OS (Falcone et al., 2007). The enhanced efficacy of FOLFOXIRI can be attributed to the addition of oxaliplatin, which synergizes with 5-fluorouracil (5-FU) and leucovorin to exert potent antitumor effects (Frerker et al., 2023). Oxaliplatin forms platinum-DNA adducts that lead to DNA damage and apoptosis, while 5-FU inhibits thymidylate synthase, an enzyme necessary for DNA synthesis (Vaughn et al., 2020; Pranzini et al., 2022). This multi-targeted approach amplifies cytotoxic effects on cancer cells, potentially creating a more favorable environment for CET maintenance therapy to exert its benefits. Moreover, CET may overcome resistance to oxaliplatin by blocking EGFR-mediated signaling, thereby enhancing the cytotoxic effects of chemotherapy and restoring sensitivity to treatment (Chen P. et al., 2020). This rationale aligns with the current European Society for Medical Oncology (ESMO) guidelines, which recommended maintenance treatment with fluoropyrimidine plus CET after oxaliplatin-based chemotherapy (CervantesA et al., 2023). On the other hand, a retrospective analysis of the TRIBE trial by GONO indicated that in left-sided tumors, the benefits of intensifying the FOLFOXIRI may be less pronounced (Cremolini et al., 2018b). In such cases, FOLFIRI combined with an anti-EGFR agent like CET emerges as a preferred option. Additionally, FOLFIRI may be a more suitable choice for patients who cannot tolerate the higher toxicity associated with FOLFOXIRI or when a less intensive regimen is clinically warranted.

Both CET monotherapy maintenance and combination maintenance therapy provided patients with PFS benefit. Admittedly, the debate over whether CET monotherapy or combination therapy is preferable remains controversial. Although the ERMES (Pinto et al., 2024) phase 3 trial did not demonstrate noninferiority of maintenance with CET monotherapy compared with FOLFIRI plus CET, results from MACRO-2 (Aranda et al., 2018) have suggested that PFS, OS and safety profile were similar between CET alone and FOLFOX + CET, indicating that CET alone may be a viable maintenance therapy option in mCRC patients. This disparity highlights the need for additional research to clarify the optimal approach for maintaining treatment efficacy.

CET maintenance demonstrated improvements in both PFS and OS when compared with observation, whereas no significant survival difference was observed when bevacizumab was used as the control arm. This is clinically understandable, as maintenance treatment is expected to perform better than treatment discontinuation, while the relative benefit between two active maintenance strategies may be less pronounced. The efficacy of bevacizumab maintenance itself is supported by the phase III CAIRO3 study, which showed that capecitabine plus bevacizumab maintenance after capecitabine, oxaliplatin, and bevacizumab induction was effective in first-line mCRC treatment across molecular subgroups (Goey et al., 2017). Therefore, the lack of significant difference between CET and bevacizumab maintenance in our analysis should not necessarily be interpreted as an absence of CET activity, but rather may reflect the fact that both agents provide meaningful disease control in the maintenance setting.

Subgroup analysis has also suggested that mCRC patients from Asia were more likely to receive OS benefits when treated with CET maintenance. In contrast, no discernible OS benefits from CET maintenance were observed in mCRC patients from Europe. This variability in the effects of CET among different races and regions could be attributed to genetic predisposition. Research has shown that KRAS/BRAF wild-type tumors were most common among Asians (66.7%), and the frequency differed compared with tumors from white people(s) (51.2%) (Yoon et al., 2015). KRAS is a key downstream effector of the EGFR signaling pathway. KRAS mutations can isolate the pathway from the effect of EGFR2, thus rendering CET ineffective in inhibiting tumor growth (Karapetis et al., 2008). Since mCRC patients who have KRAS wild-type tumors are more likely to respond to treatment with CET, it may be reasonable to assume that the Asians were more likely to receive OS benefits from CET maintenance. Similar result was found in ASPECCT trial which compared the efficacy and safety of panitumumab and CET in patients with chemotherapy-refractory mCRC (Price et al., 2014). The HR for PFS in Asian patients was 1.17 (95% CI 0.97–1.42), and for White patients, it was 0.92 (95% CI 0.77–1.09). These findings suggested a trend towards longer PFS with CET in Asian patients. However, it should be interpreted with care because the trend was not statistically significant.

It is noteworthy that the survival benefit provided by CET maintenance may vary among individual with different primary tumor site and metastases site. A study has shown that the median overall survival (mOS) from the use of CET maintenance was better in patients with only lung metastases compared with those with only liver metastases (Yuan et al., 2021). Similarly, the recent EPOC study did not support the addition of CET to chemotherapy for patients with operable colorectal liver metastases (Katipally et al., 2023). This discrepancy in efficacy may be attributed to differences in the molecular profiles of lung metastases and liver metastases. Lung tumors, particularly non-small cell lung cancers (NSCLC), generally exhibit higher EGFR expression compared to liver tumors (Levantini et al., 2022). Since CET specifically targets EGFR, tumors with elevated EGFR expression are more likely to respond to CET treatment. Moreover, liver metastases can impact liver function and lead to complications such as liver failure or impaired drug metabolism, which may limit treatment options and worsen prognosis (Primrose, 2010). In contrast, lung metastases may have less direct impact on organ function, allowing for more aggressive treatment approaches. The primary tumor site also affected the OS, with left side better than right side (Yuan et al., 2021). One reason is that tumors in the left colon are less likely to harbor genetic mutations, such as KRAS mutations (Gaedcke et al., 2010). As the presence of KRAS mutations is associated with resistance to CET treatment, patients with left colon tumors are more likely to benefit from CET therapy. However, the number of patients was insufficient and further large-scale studies are needed to investigate the appropriateness.

The overall safety of CET maintenance is acceptable as it did not increase the risk of most AEs involved in the study, such as anemia, nausea, vomiting, mucositis and fatigue, irrespective of the severity. This advantage may alleviate pain, discomfort and depression caused by these symptoms, and mitigate the dangers of dehydration, hypoxia, and malnutrition. Nevertheless, it is important to note that CET maintenance does give rise to certain AEs that warrant attention. When hematopoietic effects were concerned, neutropenia was significantly predisposed to occur in high-grade but not all-grade neutropenia, aligning with the findings by Sclafani et al. (Abdel-Rahman and Fouad, 2015). Infections in neutropenic individuals can be severe, progress rapidly, and may be more challenging to treat. Therefore, monitoring of hematologic parameters after medication administration is important. CET maintenance also increased the incidence of all-grade diarrhea, but not across high grade. This observation parallels findings in a prospective study, which also revealed a connection between the severity of diarrhea and the dose of CET (Reis et al., 2015). Therefore, exploring low-dose fractional administration may be a viable approach to mitigate the impact on diarrhea. Skin toxicity is the most common adverse effects of CET. In line with earlier research findings, our study demonstrated that CET maintenance increased the incidence of rash in all grade. However, it is noteworthy that this rash can be effectively controlled using standard topical and systemic therapies. Intriguingly, various clinical trials have shown that mCRC patients treated with CET, who developed skin rash, exhibited improved PFS and OS compared with those without such skin reactions (Stintzing et al., 2013; Jonker et al., 2007). This underscores the possibility that CET-induced skin rash might serve as a prognostic factor in mCRC patients. Overall, the AEs associated with CET maintenance are manageable but there is a need for improvement through low-dose fractional administration under multidisciplinary monitoring.

Several limitations of this meta-analysis should be acknowledged. First, the control regimens differed substantially across the included studies, including observation, bevacizumab, and capecitabine-based maintenance strategies. Such heterogeneity in comparator arms may influence the pooled effect estimates and limit the direct comparability of survival outcomes between studies. In particular, CET maintenance showed clearer benefit when compared with observation, whereas no significant difference was observed versus bevacizumab, suggesting that the choice of control regimen may substantially affect the interpretation of treatment efficacy. Second, due to the limited number of eligible studies, both randomized controlled trials and cohort studies were included to provide more comprehensive evidence. Although subgroup analyses according to study design were performed, potential selection bias and methodological heterogeneity cannot be completely excluded. Third, the funnel plot for the primary outcome demonstrated a certain degree of asymmetry, which may indicate potential publication bias. However, given the small number of included studies, this finding should be interpreted cautiously, as asymmetry may also reflect methodological heterogeneity or inflated effect sizes in smaller studies. Fourth, meta-regression could not be conducted because of the limited number of studies available. In addition, the relatively small overall sample size and limited number of studies in several subgroup analyses may reduce statistical power and restrict the generalizability of the findings. Therefore, further large-scale prospective studies with standardized control arms are warranted to validate these results. Moreover, limited data on ORR prevented further pooled analysis of tumor response outcomes.

## Conclusion

In general, CET maintenance therapy may be associated with better OS and PFS outcomes compared with observation in patients with mCRC, with a more pronounced OS benefit observed in Asian populations. Although CET maintenance increased the incidence of diarrhea and rash, the overall toxicity profile was generally manageable. Nevertheless, the limited number of included studies and patients may affect the robustness of the findings. Therefore, further large-scale, multicenter randomized controlled trials are warranted to validate these results.

## Data Availability

The raw data supporting the conclusions of this article will be made available by the authors, without undue reservation.
