# Supplementary figures and images for: Old tale new admirers, cetuximab maintenance in metastatic colorectal cancer: a systematic review and meta-analysis
Source: Front Pharmacol. 2026 Jun 3;17:1845800. doi: 10.3389/fphar.2026.1845800 (PMC13272484; doi:10.3389/fphar.2026.1845800)

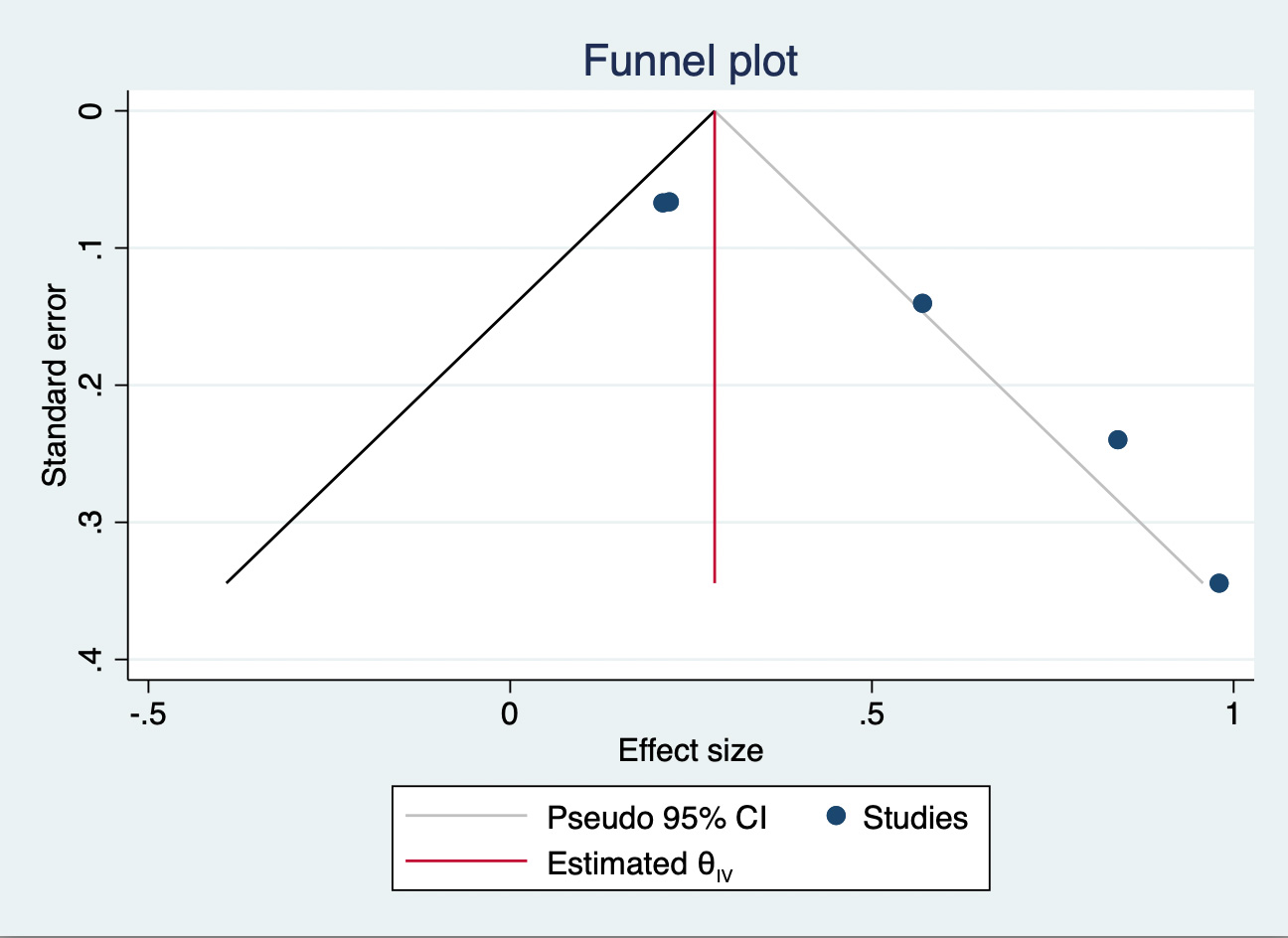

Supplement: Supplementary file 1 [file Image3.jpeg]

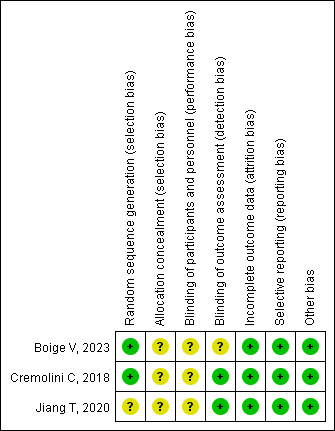

Supplement: Supplementary file 3 [file Image1.jpeg]

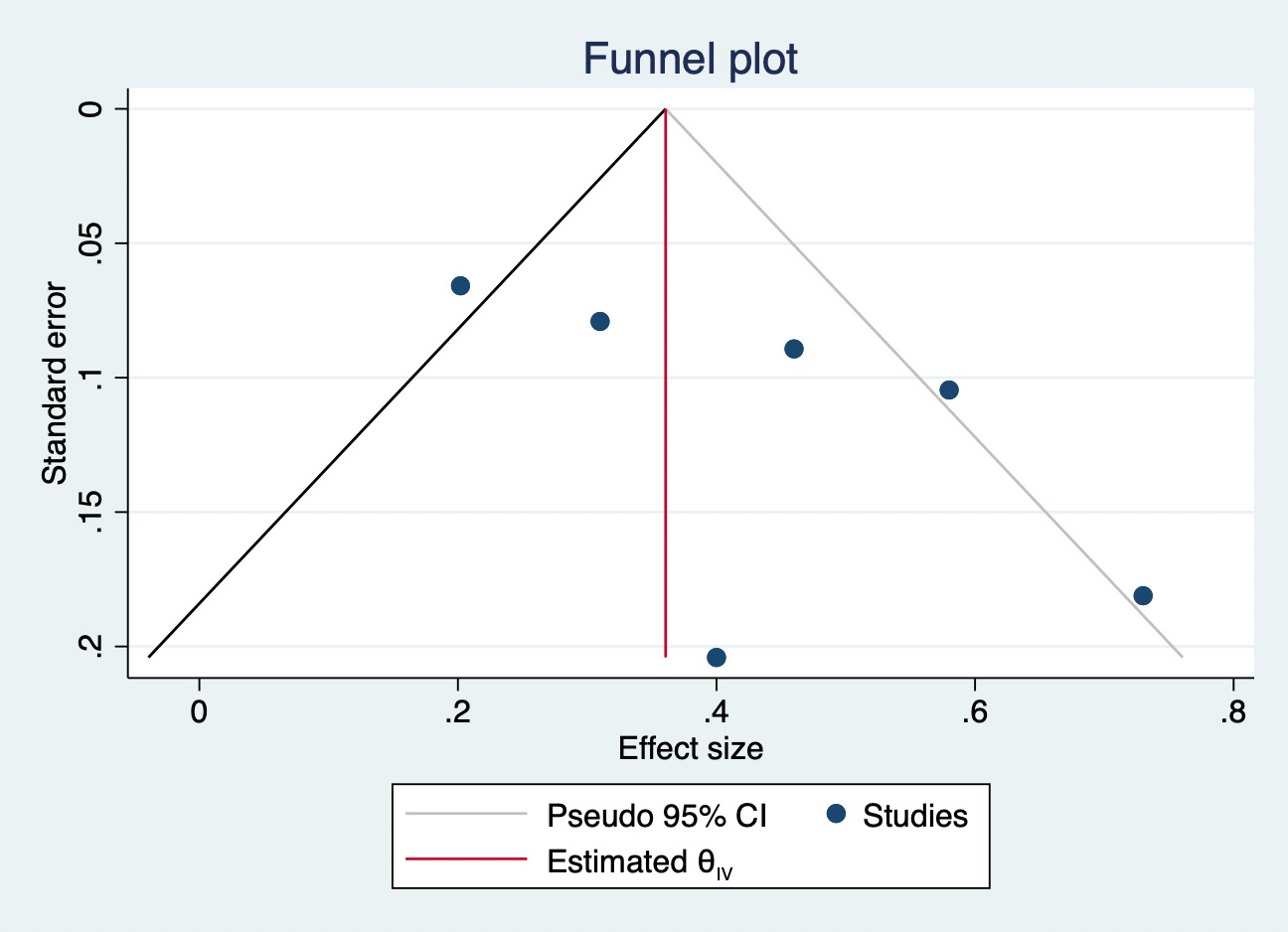

Supplement: Supplementary file 4 [file Image4.jpeg]

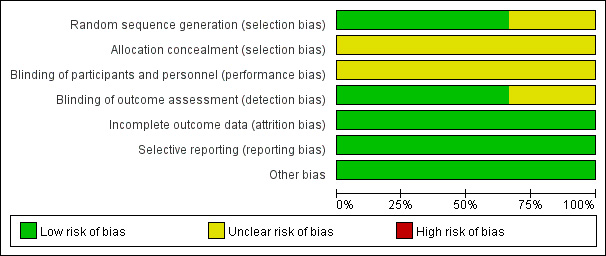

Supplement: Supplementary file 5 [file Image2.jpeg]

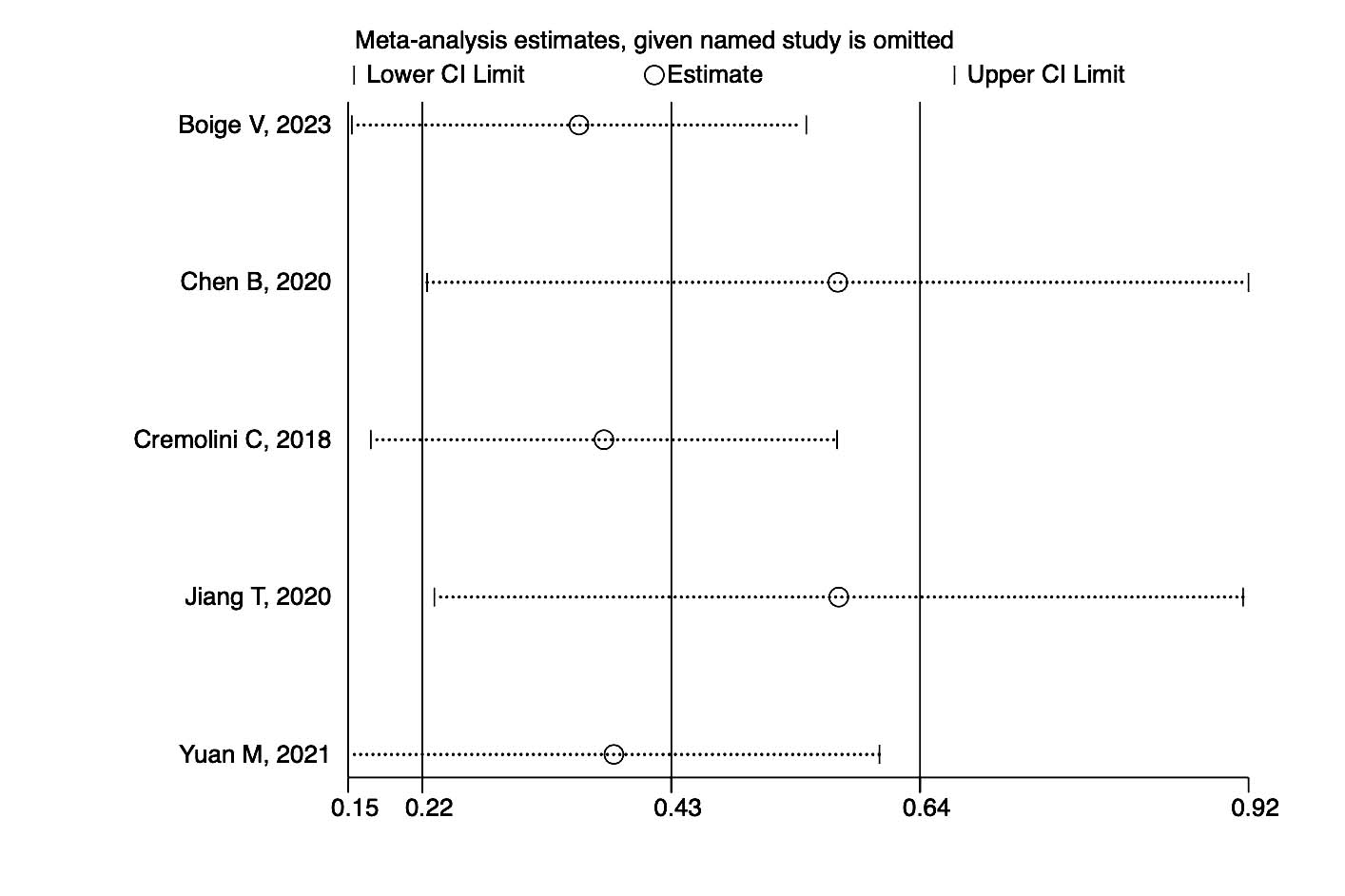

Supplement: Supplementary file 6 [file Image5.jpeg]

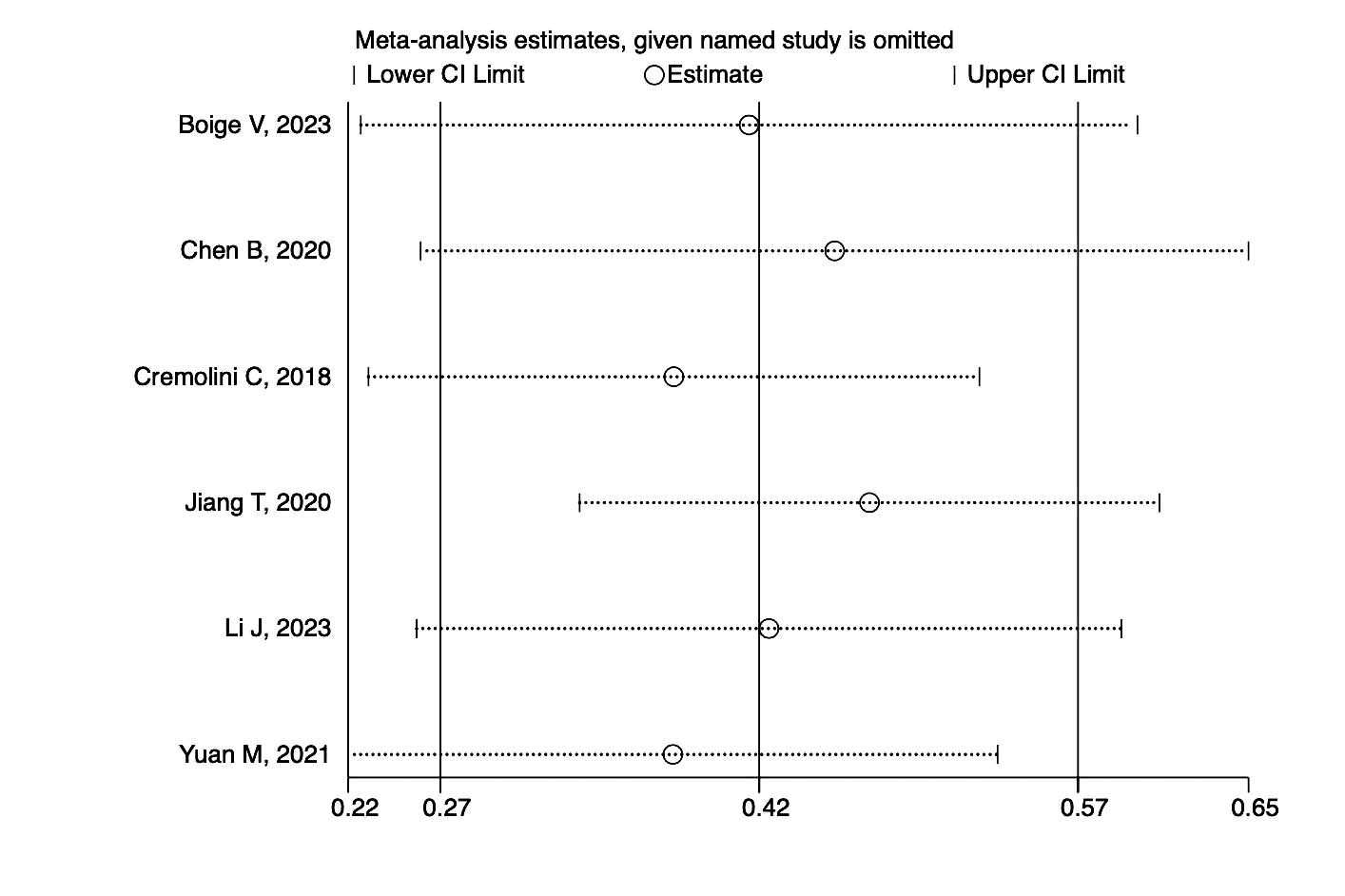

Supplement: Supplementary file 10 [file Image6.jpeg]
