# Supplementary material for: Old tale new admirers, cetuximab maintenance in metastatic colorectal cancer: a systematic review and meta-analysis
Source: Front Pharmacol. 2026 Jun 3;17:1845800. doi: 10.3389/fphar.2026.1845800 (PMC13272484; doi:10.3389/fphar.2026.1845800)
Supplement: Supplementary file 2 [file Table1.docx]

**Supplementary Table 1. Search Strategy.**

**Data searched from PubMed**

| Search Query | Results |
| --- | --- |
| #1 ("Colorectal Neoplasms"[Mesh]) OR "colorectal cancer*" OR "colorectal tumor*" OR "colorectal neoplasm*" | 285,880 |
| #2 (Cetuximab[MeSH]) OR Erbitux OR IMC C225 OR IMC-C225 OR MAb C225 | 9,002 |
| #3 #1 AND #2 | 3,788 |

Searched before March, 2026. Results: 3,788.

**Data searched from** **Embase**

| Search Query | Results |
| --- | --- |
| #1 'colorectal cancer'/exp OR 'colorectal tumor*':ti,ab,kw OR 'colorectal neoplasm*':ti,ab,kw | 402,662 |
| #2 'cetuximab'/exp OR 'erbitux':ti,ab,kw | 34,661 |
| #3 #1 AND #2 | 5629 |

Searched before March, 2026. Results: 14,799.

**Data searched from Cochrane Library**

| Search Query | Results |
| --- | --- |
| #1 MeSH descriptor: [Colorectal Neoplasms] explode all trees OR (“colorectal cancer”):ti,ab,kw OR (“colorectal neoplasm”):ti,ab,kw OR (“colorectal tumor”):ti,ab,kw OR (“colorectal cancers”):ti,ab,kw OR (“colorectal tumors”):ti,ab,kw OR (“colorectal neoplasms”):ti,ab,kw | 21,034 |
| #2 MeSH descriptor: [Cetuximab] explode all trees OR (Erbitux):ti,ab,kw | 999 |
| #3 #1 AND #2 | 516 |

Searched before March, 2026. Results: 516.

**Data searched from Scopus**

| Search Query | Results |
| --- | --- |
| #1 "colorectal neoplasm" OR "colorectal cancer" OR "colorectal tumor" | 238,511 |
| #2 "Cetuximab" OR "Erbitux" | 28,710 |
| #3 #1 AND #2 | 5,238 |

Searched before March, 2026. Results: 5,238.
