# Supplementary material for: Old tale new admirers, cetuximab maintenance in metastatic colorectal cancer: a systematic review and meta-analysis
Source: Front Pharmacol. 2026 Jun 3;17:1845800. doi: 10.3389/fphar.2026.1845800 (PMC13272484; doi:10.3389/fphar.2026.1845800)
Supplement: Supplementary file 7 [file Table2.docx]

**Supplementary Table 2. Further Characteristics of the included trials.**

| Author, year | Median age, yrs | Masking | Phase | Source of population | RAS status | Confounders | Intervene | | | Control | | |
| --- | --- | --- | --- | --- | --- | --- | --- | --- | --- | --- | --- | --- |
|  |  |  |  |  |  |  | No. of patients | Median PFS, mo | Median OS,  mo | No. of patients | Median PFS, mo | Median OS,  mo |
| Boige V, 2023 | 67.0 | Open-label | 2 | NA | wild-type | NA | 67 | 5.3  (3.7-7.4) | 24.8  (18.7-30.4) | 72 | 2.0  (1.8-2.7) | 19.7  (13.3-24.4) |
| Chen B, 2020 | NA | NA | NA | Hospital database | wild-type | age,primary tumor site, site of specimen, duration of treatment, ECOG PS, number of metastatic sites, response  to prior induction treatment, and time from induction treatment to start | 110 | 21.9  (16.4-24.4) | 26.0  (23.4-28.7) | 112 | 17.7  (11.3-19.0) | 22.7  (21.2-24.3) |
| Cremolini C, 2018 | 59.5 | Open-label | 2 | NA | wild-type | NA | 37 | 13.3  (11.2-17.3) | 37.5  (32.0-NE) | 41 | 10.8  (9.3-13.9) | 37.0  (30.0-NE) |
| Jiang T, 2020 | NA | NA | NA | NA | wild-type | NA | 44 | 6.1  (6.0-6.2) | NA | 28 | NA | NA |
| Li J,2023 | NA | NA | NA | Hospital database | wild-type | sex, age, primary tumor site, PS score, primary tumor resection, matastasis time, degree of differentiation, maintenance baseline CEA, MSI status, number of metastatic sites, number of induction treatment cycles, and best response to induction therapy | 18 | 7.3  (5.8-8.8) | NA | 21 | NA | NA |
| Yuan M, 2021 | 52.3 | NA | NA | Hospital database | wild-type | sex, age, primary tumor site,  primary tumor resection,  and organs with metastases | 82 | NA | NA | 95 | NA | NA |

yrs, years; NA, Not Available; No, number; PFS, Progression-Free Survival; mo, month; OS, Overall Survival; NE, not estimable.
