# Supplementary material for: Old tale new admirers, cetuximab maintenance in metastatic colorectal cancer: a systematic review and meta-analysis
Source: Front Pharmacol. 2026 Jun 3;17:1845800. doi: 10.3389/fphar.2026.1845800 (PMC13272484; doi:10.3389/fphar.2026.1845800)
Supplement: Supplementary file 8 [file Table3.docx]

**Supplementary Table 3. Methodological quality of cohort studies included in the meta-analysis^*^**

| **First author,**  **publication year**  **(reference)** | **Selection** | | | | **Comparability** | **Outcome** | | | **Total score** |
| --- | --- | --- | --- | --- | --- | --- | --- | --- | --- |
|  | **Representativeness of the exposed cohort** | **Selection of the unexposed**  **cohort** | **Ascertainment**  **of exposure** | **Outcome of interest not present at start of study** | **Control for**  **important factor or additional factor†** | **Assessment of outcome** | **Follow-up**  **long enough for outcomes**  **to occur ‡** | **Adequacy of**  **follow-up**  **of cohorts §** |  |
| Chen B, 2020 | ⚝ | ⚝ | ⚝ | - | ⚝ | ⚝ | - | - | 6 |
| Li J, 2023 | ⚝ | ⚝ | ⚝ | ⚝ | - | - | - | ⚝ | 5 |
| Yuan M, 2021 | ⚝ | ⚝ | ⚝ | - | - | - | - | ⚝ | 4 |

* A study could be awarded a maximum of one star for each item except for the item Control for important factor or additional factor.

† A maximum of 2 stars could be awarded for this item. Studies that controlled for sex, race, area, calendar year, period (5-year groups), socioeconomic status, comorbidities, cancer site, cancer type, incidence rate, and mortality received two stars.

‡ A cohort study with a follow-up time >5 y was assigned one star.

§ A cohort study with a follow-up rate >90% was assigned one star
