# Supplementary material for: Old tale new admirers, cetuximab maintenance in metastatic colorectal cancer: a systematic review and meta-analysis
Source: Front Pharmacol. 2026 Jun 3;17:1845800. doi: 10.3389/fphar.2026.1845800 (PMC13272484; doi:10.3389/fphar.2026.1845800)
Supplement: Supplementary file 9 [file Table4.docx]

**Supplementary Table 4.** Quality assessment of the evidence of the included publications, based on the GRADE

| **Quality assessment** | | | | | | | **No of patients** | | **Effect** | | **Quality** | **Importance** |  |
| --- | --- | --- | --- | --- | --- | --- | --- | --- | --- | --- | --- | --- | --- |
|  | | | | | | |  | |  | |  |  |  |
| **No of studies** | **Design** | **Risk of bias** | **Inconsistency** | **Indirectness** | **Imprecision** | **Other considerations** | **Intervention** | **Control** | **Relative (95% CI)** | **Absolute** |  |  |  |
| **PFS** | | | | | | | | | | | | |  |
| 6 | Mixed design | not serious | serious | not serious | not serious | none | 358 | 369 | HR:0.42  (0.27,0.57) | 107/500 | ○ MODERATE | IMPORTANT |  |
|  |  |  |  |  |  |  |  |  |  |  |  |  |  |
| **OS** | | | | | | | | | | | | |  |
| 5 | Mixed design | not serious | serious | not serious | not serious | none | 340 | 348 | HR:0.47 (0.19,0.74) | 96/299 | ○  MODERATE | IMPORTANT |  |

No, numbers; CI, confidence interval; PFS, progress-free survival; OS, overall survival; HR, hazard ratio.
